# Comparisons of Copy Number, Genomic Structure, and Conserved Motifs for α-Amylase Genes from Barley, Rice, and Wheat

**DOI:** 10.3389/fpls.2017.01727

**Published:** 2017-10-05

**Authors:** Qisen Zhang, Chengdao Li

**Affiliations:** ^1^Australian Export Grains Innovation Centre, South Perth, WA, Australia; ^2^Western Barley Genetics Alliance, Murdoch University, Murdoch, WA, Australia

**Keywords:** α-amylase, barley, conserved motif, genome, gibberellin responsive complex, promoter

## Abstract

Barley is an important crop for the production of malt and beer. However, crops such as rice and wheat are rarely used for malting. α-amylase is the key enzyme that degrades starch during malting. In this study, we compared the genomic properties, gene copies, and conserved promoter motifs of α-amylase genes in barley, rice, and wheat. In all three crops, α-amylase consists of four subfamilies designated *amy1, amy2*, *amy3*, and *amy4*. In wheat and barley, members of *amy1* and *amy2* genes are localized on chromosomes 6 and 7, respectively. In rice, members of *amy1* genes are found on chromosomes 1 and 2, and *amy2* genes on chromosome 6. The barley genome has six *amy1* members and three *amy2* members. The wheat B genome contains four *amy1* members and three *amy2* members, while the rice genome has three *amy1* members and one *amy2* member. The B genome has mostly *amy1* and *amy2* members among the three wheat genomes. *Amy1* promoters from all three crop genomes contain a GA-responsive complex consisting of a GA-responsive element (CAATAAA), pyrimidine box (CCTTTT) and TATCCAT/C box. This study has shown that *amy1* and *amy2* from both wheat and barley have similar genomic properties, including exon/intron structures and GA-responsive elements on promoters, but these differ in rice. Like barley, wheat should have sufficient amy activity to degrade starch completely during malting. Other factors, such as high protein with haze issues and the lack of husk causing Lauting difficulty, may limit the use of wheat for brewing.

## Introduction

The best quality barley grains are used predominantly for making malts and subsequently beer and whiskey. Malting consists of steeping, germination, and kilning (Gupta et al., 2010). Steeping and germination allow production of hydrolyzing enzymes including α-amylase (amy), β-amylase, limit dextrinase, and α-glucosidase for starch degradation (Bak-Jensen et al., 2007; Evans et al., 2010; Fincher, 2010; Shahpiri et al., 2015). Starch comprises an α-D-glucose homo-polymer amylose and branched amylopectin. The former is a linear molecule of α-1,4-linked glucose molecules, while the latter is a larger molecule with α-1,6 branching points (Bahaji et al., 2014). Amy [α-(1,4)-D-glucan glucanohydrolase, EC 3.2.1.1] cleaves α-(1,4) glycosidic linkage internally to produce oligosaccharides and amylopectin. Amy is the most important enzyme for starch degradation during malting and mashing. Barley malts contain sufficient active amy enzymes to almost completely hydrolyze starch during malting and mashing.

Activation of amy expression is strictly controlled by the phytohormones gibberellin and ABA. During grain development, amy expression is repressed by ABA. However, in a genetic defect wheat, a high level of high pI amy genes could be expressed, resulting in poor grain quality during late grain development. This is normally referred to as late maturity α-amylase (LMA) (Barrero et al., 2013). During seed germination, amy expression is induced by elevated GA levels (Lanahan et al., 1992; Gómez-Cadenas et al., 2001; Woodger et al., 2010).

Genetic mapping associated barley malt amy activities with amy1 and amy2 loci on chromosomes 6H and 7H, respectively (Hayes et al., 1993; Oziel et al., 1996; Zale et al., 2000; Gao et al., 2004; Li et al., 2010). Isoelectric focusing electrophoresis identified low and high pI amy isoforms in barley aleurone extracts (Jacobsen and Higgins, 1982; Svensson et al., 1985). However, the number of amy isoforms in the barley genome is unknown but predicted to be from three to eight (Jacobsen and Higgins, 1982; Muthukrishnan et al., 1984; Svensson et al., 1985; Khursheed and Rogers, 1988; Evans et al., 2010). Nomenclatures of amys are complicated. Two families of amy, were named AMY1 and AMY2, referred to low and high pI enzymes, respectively (MacGregor et al., 1971; Jacobsen and Higgins, 1982; Svensson et al., 1985; Evans et al., 2010). A genomic clone and two cDNA clones coding for amy enzymes have been named amy32b, amy6-4, and amy46 (Rogers and Milliman, 1983; Whittier et al., 1987; Khursheed and Rogers, 1988). In a recently published barley genomic sequencing paper, new amy nomenclatures have been proposed. The barley genome contains at least 12 *amy* genes, grouped into four subfamilies *amy1*, *amy2*, *amy3*, and *amy4* (Mascher et al., 2017). Here, we compared gene copy numbers, genomic structures and promoter conserved motifs of *amy1* and *amy2* subfamilies from barley, wheat, and rice. We hypothesize that the expansion in *amy1* members combined with the presence of conserved regulatory motifs on promoters of *amy1* and *amy2* genes are important determinants for selecting barley as a malting crop.

## Materials and Methods

Genome sequences were downloaded to a local computer from ftp://ftp.ensemblgenomes.org/pub/plants/release-35/fasta/hordeum_vulgare/dna/ for barley; ftp://ftp.ensemblgenomes.org/pub/plants/release-35/fasta/oryza_sativa/dna/ for rice, and ftp://ftp.ensemblgenomes.org/pub/plants/release-35/fasta/triticum_aestivum/dna/ for wheat. The identification of barley, wheat, and rice amy genes are described in Mascher et al. (2017). *Amy* coding and promoter sequences (500 bp upstream of the translation start codon ATG) for all three crops were extracted after being blasted with the *amy* genes. Briefly, the *amy* genes were used to blast standalone blastable genomic databases to obtain *amy* gene nucleotide positions in pseudomolecules. According to these positions, the *amy* gene coding and promoter sequences were calculated and extracted with a Perl script. The promoter sequences were aligned with a ClustalW program^1^ and conserved motifs were examined.

## Results and Discussion

### Barley *amy* Copy Numbers – Historical and Genomic Evidence

Barley *amy* genes were initially mapped to chromosomes 6H and 7H with wheat–barley addition lines (Brown and Jacobsen, 1982; Muthukrishnan et al., 1984). Southern blot analysis of two different *amy* gene DNA probes detected at least six and three hybridization bands from addition lines containing barley chromosomes 6H and 7H, respectively (Muthukrishnan et al., 1984; Rogers and Milliman, 1984). There were multiple amy protein bands on SDS PAGE purified from the barley aleurone using cycloheptaamylose-sepharose affinity chromatography and at least four amy activity peaks separated by DEAE cellulose chromatography (Jacobsen and Higgins, 1982). These offered early experimental evidence of the *amy* multigene family. Isoelectric focusing (IEF) electrophoresis showed that purified amy proteins could be divided into two distinct groups, a low pI group with an isoelectric point of 4.5–5.1 and a high pI group with an isoelectric point of 5.0–6.6 (Jacobsen and Higgins, 1982). Due to its commercial and biological importance, a significant effort was made to clone *amy* genes. A genomic clone was identified as an *amy* gene (amy32b) and belongs to a low pI amy protein (Rogers and Milliman, 1983; Whittier et al., 1987). Two cDNA clones were also characterized as *amy* genes (amy6_4 and amy46) that belong to high pI enzymes (Khursheed and Rogers, 1988). Furthermore, 3D structures have been resolved for two barley amy proteins; one belonging to a low pI amy protein (1AMY) and the other to a high pI amy protein (1HT6) (Kadziola et al., 1994; Robert et al., 2003).

While experimental data has shown that amy proteins are coded by multigene families, the exact numbers of genes are unknown. Barley genome sequencing is a useful resource for identifying the number of amy genes and discovering their genomic features. The barley genome contained 12 *amy* genes (Mascher et al., 2017), which were grouped into four subfamilies (**Table 1**). Subfamily 1 consists of six members—four on chromosome 6H (533880485–542858990 bp) and two on the unsorted chromosome (195047130–196261798 bp, **Table 1**)—designated *amy1_1a* to *amy1_1e* and *amy1_2*. Four of which (*amy1_1a* to *amy1_1d*) have almost 100% sequence identity among members (Additional File 1: Supplementary Table S1A). One member (*amy1_1e*) is a truncated protein missing the carbohydrate-binding domain. Sequence identity analysis showed that the five *amy1_1* proteins matched the cloned gene *amy6_4* (Khursheed and Rogers, 1988), while the *amy1_2* protein, with 95% sequence identity with *amy1_1a*, matched the cloned gene amy46 in both promoter and coding regions (Khursheed and Rogers, 1988). All amy1 members belong to high pI enzymes (**Table 1**) and have high sequence identity with a 3D structure-resolved protein 1AMY (Kadziola et al., 1994). Subfamily *amy2* has three members on chromosome 7H (556169683–557427021 bp, **Table 1**), and are designated *amy2_1* to *amy2_3*. They have >92% sequence identity among the members, and >72% when compared to *amy1_1a*. *Amy2_3* had a high sequence identity with cloned gene *amy32b* (Rogers, 1985; Whittier et al., 1987) and 3D structure-resolved protein 1HT6 (Robert et al., 2003). They belong to genes coding for low pI enzymes (**Table 1**). *Amy3* has one member localized on chromosome 5H (designated *amy3*), while *amy4* has two members localized on chromosomes 2H and 3H (designated *amy4_1* and *amy4_2*). The *amy4* members have about 48% sequence identities compared between the members, or 43–46% sequence identity when compared to *amy1_1a* (Supplementary Table S1A).

**Table 1:** Barley amy nomenclatures, gene ID, locations, and the association of old and new nomenclatures.

New names	IBSC gene ID	Chr	Genomic location	SF	Old nomenclatures
amy1_1a	HORVU6Hr1G078330	6H	533880485/533879015	1	*amy6_4, 1AMY, high pI*
amy1_1b	HORVU6Hr1G078360	6H	534112867/534114337	1	*amy6_4, 1AMY, high pI*
amy1_1c	HORVU6Hr1G078420	6H	534499529/534498059	1	*amy6_4, 1AMY, high pI*
amy1_1d	HORVU0Hr1G032700	0H	195047130/195048600	1	*amy6_4, 1AMY, high pI*
amy1_1e	HORVU0Hr1G032850	0H	196262594/196261798	1	*amy6_4, 1AMY, high pI*
amy1_2	HORVU6Hr1G080790	6H	542857506/542858990	1	*amy46, 1AMY, high pI*
amy2_1	HORVU7Hr1G091150	7H	556169683/556167920	2	low pI
amy2_2	HORVU7Hr1G091240	7H	557398785/557397068	2	low pI
amy2_3	HORVU7Hr1G091250	7H	557428810/557427021	2	*amy32b, 1HT6, low pI*
amy3	HORVU5Hr1G068350	5H	517452674/517454307	3	N/A
amy4_1	HORVU2Hr1G071710	2H	511664000/511667683	4	N/A
amy4_2	HORVU3Hr1G067620	3H	513498473/513485531	4	N/A

*Chr, chromosome; SF, subfamily*.

Since *amy1* and *amy2* were located on chromosomes 6H and 7H, respectively, and many important malt quality QTLs were associated with these genetic loci, we believed that they were the most important members in relation to barley malt qualities (Hayes et al., 1993; Oziel et al., 1996; Zale et al., 2000; Gao et al., 2004; Li et al., 2010), we decided to investigate and compare gene and promoter structures for these two subfamilies in barley, rice, and wheat.

### Barley amy Protein Secondary Structure

**FIGURE 1:**
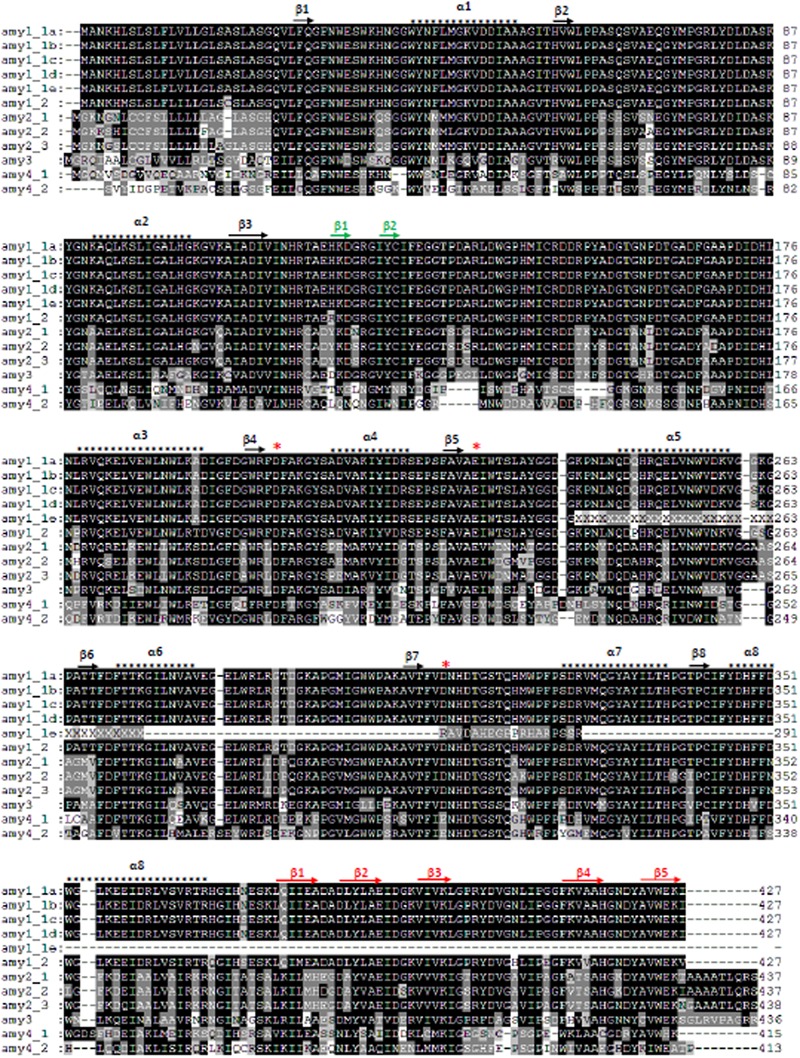
Alignments and secondary structures of barley amy proteins. Barley amy1_1a and amy2_3 had 100% sequence identities with the two 3D structure-resolved barley amy proteins (1AMY and 1HT6), respectively (Kadziola et al., 1994; Robert et al., 2003). Their secondary structure features are shown on top of the alignments. There are three domains: domain A (black arrows and asterisks), domain B (green arrows), and domain C (red arrows). Domain A consists of a (α/β)_8_ barrel, while domain C has five β-sheets. The three catalytic amino acids Asp_203_, Glu_228_, and Asp_310_ are indicated by red asterisks (amy1_1a position).

All amy proteins from amy1 and amy2 had the catalytic amino acid residues Asp_203_, Glu_228_, and Asp_310_ (*amy1_1a* positions), apart from *amy1_1e*, which was a truncated protein missing Asp_310_ (**Figure 1**). The near full-length proteins consisted of a central domain A forming (α/β)_8_ barrel, a structural loop domain B and a carbohydrate-binding domain C (**Figure 1**). Domain C formed five anti-parallel sheets (Kadziola et al., 1994; Robert et al., 2003). Barley amy proteins from amy3 and amy4 also contain the catalytic amino acids and a carbohydrate-binding module as discovered in the Domain Database at NCBI^2^. However, Asp_310_ on the active site was replaced with Glu_310_ for the two amy4 proteins (**Figure 1**).

### Barley *amy* Gene Genomic Arrangement

Four barley *amy1* genes (*amy1_1a* to *amy1_1d*) had the same genomic arrangements as two introns and three exons. The nucleotide numbers for the introns and exons were the same as *amy1_1a* to *amy1_1d*, being 23 and 107 bp for the introns and 87, 1002, and 252 bp for the exons (Supplementary Table S2). While *amy1_2* had two introns and three exons, the nucleotide numbers differed from the *amy1_1a* to *amy1_1d* members, being 95 and 106 bp for the introns and 87, 945, and 252 bp for the exons. *Amy2-1* to *amy2_3* had three introns and four exons, but the nucleotide numbers for all introns and exons differed among the three *amy* genes (Supplementary Table S2). *Amy3* had three introns and four exons like *amy2*, but the nucleotide numbers differed from *amy2*. *Amy4* had more than five introns and six exons.

### Barley *amy* Gene Promoter Conserved Motifs

**FIGURE 2:**
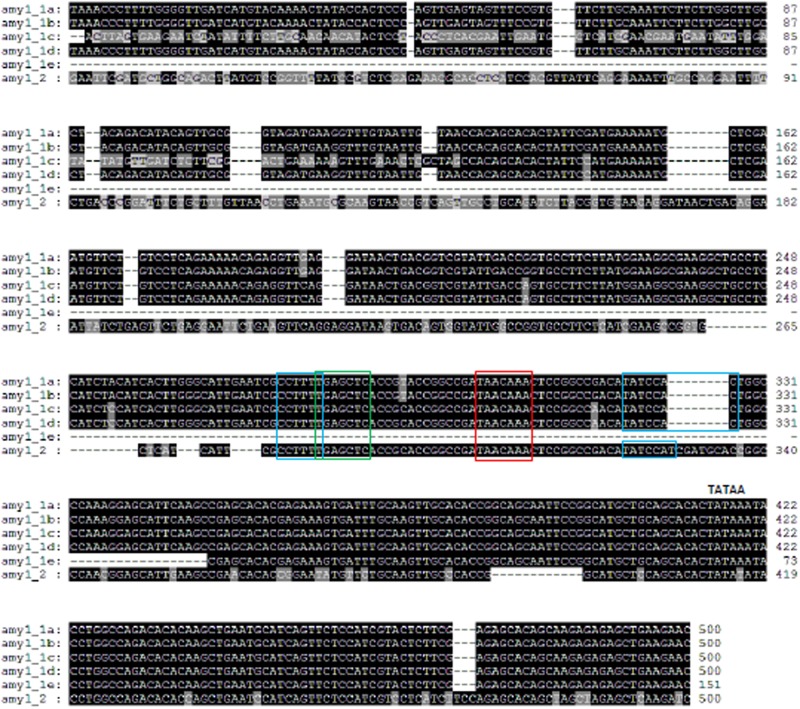
Barley *amy1* promoter sequence alignments and conserved motifs. ALL *amy1* promoters (except *amy1_1e*, which is truncated) contain a GA-responsive element (GARE) TAACAAA (red box). It requires for GA induction, They also contain a pyrimidine box (CCTTTT) and a TATCCA(C/T) box (blue boxes), which enhance gene expression after GA responses (Gubler and Jacobsen, 1992). A cAMP-like responsive element (TGAGCTC) is conserved (green box).

**FIGURE 3:**
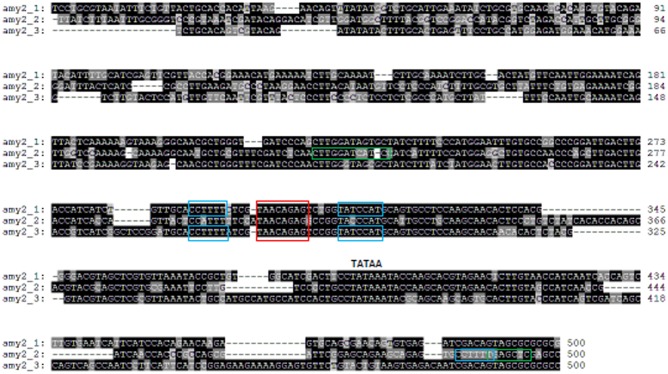
Barley *amy2* promoter sequence alignments and conserved motif analysis. All three *amy2* promoters contain a GA-responsive element (GARE) TAACAGAG (red box) required for GA induction. Promoters of *amy2_1* and *amy2_3* contained a pyrimidine box (CCTTTT) and a TATCCA(C/T) box (blue boxes), which enhance gene expression (Gubler and Jacobsen, 1992). A pyrimidine box (blue box), a cAMP-like responsive element (TGAGCTC) (green box) and an O2S (CTTGXXTCATC) (green box) were present on *amy2_2* promoter.

Promoters of all barley *amy1* genes contained a conserved GA response complex (GARC) consisting of GARE (TAACAAA), pyrimidine (CCTTTT) and TATCCAC(T) boxes (Supplementary Table S3A and **Figure 2**) (Skriver et al., 1991; Gubler and Jacobsen, 1992; Rogers et al., 1994). There was also a cAMP-like response element (TGAGCTC) on *amy1* promoters (Gubler and Jacobsen, 1992), which represses gibberellin action. Pyrimidine and TATCCAC boxes enhanced the expression of amy1 proteins. The conserved motifs on subfamily 2 members differed from those on subfamily 1 genes and also among subfamily 2 members (Supplementary Table S3B and **Figure 3**). All three *amy2* genes had GARE (TAACAGAG) and pyrimidine (CCTTTT) boxes. The pyrimidine box was much closer to the translation start site (-17 bp) for *amy2_2*, but further away for *amy2_1*, and *amy2_3* at -211 and -236 bp, respectively. The original pyrimidine box of the *amy2_2* gene, at a similar position to *amy2_1*, and *amy2_3*, was mutated to CCATTT on *amy2_2* (**Figure 3**). A TATCCAT box was found in two *amy2* genes (*amy2_1* and *amy2_3*), but it was replaced with TACCCAT in the *amy2_2* gene. Furthermore, the *amy2_2* promoter had a conserved O2S box (CTTGxxTCATC) and cAMP-like box (TGAGCTC). Genomic sequence analysis showed that both *amy1* and *amy2* genes had a GARC, where GARE was required for GA induction of amy expression with pyrimidine and TA(T/C)CCAT box controlling gene expression levels (Lanahan et al., 1992).

When promoter regions (-500 bp) of subfamilies 1 and 2 were analyzed, the sequence identities were high (>99%) among *amy1_1a*, *amy1_1b*, and *amy1_1d* (Supplementary Table S4A). However, the *amy1_1e* promoter region was truncated to -151 bp, despite being 100% identical to the promoter sequence of *amy1_1a* gene. The promoter region of *amy1_1c* had high sequence identity within -350 bp, but low sequence identity beyond -350 bp, compared to *amy1_1a* (**Figure 2**). The sequence of the *amy1_2* promoter (-500 bp) was 64% identical to the *amy1_1a* promoter. The genomic locations of the conserved motifs in *amy1* were the same for all *amy1_1* members at -180, -199, -220, and -225 bp for TATCCA, GARE, cAMY-like and pyrimidine boxes, respectively (Supplementary Table S3A). However, the locations of these motifs from the *amy1_2* gene was a nucleotide closer to the ATG translation start site compared to the locations of *amy1_1* members (Supplementary Table S3A). The sequences of *amy2* promoters had low sequence identity (59–68%) compared to their members (Supplementary Table S4B).

### Rice *amy* Gene Numbers and Conserved Motifs on Promoters

The rice genome contained 10 *amy* genes with three, one, four, and two members in subfamilies 1, 2, 3, and 4, respectively (**Table 2**). The number of *amy1* and *amy2* genes in rice was four, much less than the sum of *amy1* and *amy2* in barley. Rice *amy3* had the most gene members. Two of the *amy1* genes (LOC_Os02g52700 and LOC_OS02g52710) had three introns (Supplementary Table S2), unlike the barley *amy1* genes, which all had two introns. The other *amy1* gene (LOC_Os01g25510) had two introns. Alignment of the three amy1 protein sequences identified one protein (LOC_Os01g25510) with a very low sequence identity (24–30%) compared to the other two amy proteins (LOC_Os02g52700 and LOC_Os02g52710) (Supplementary Table S1B and **Figure S1**). The two amy1 proteins and one amy2 protein had similar secondary structures to the barley amy proteins. However, the amino acid compositions differed substantially on most of the β-strains and α-helices (**Supplementary Figure S1**). Rice had the same catalytic amino acids (Aps, Glu, and Asp) as barley. The promoters of the three rice *amy1* genes had >65% sequence identities (Supplementary Table S4C). They contained the GARE (TAACAAA), pyrimidine (CCTTTT) and TATCCAT boxes (Supplementary Table S3C and **Figure S2A**) but not the cAMY-like box. The rice amy2 protein (LOC_Os06g49970) had 72% sequence identity compared to the two amy1 proteins (LOC_OS02g527100 and LOC_Os02g52710). However, the promoter of the rice *amy2* gene only contained GARE (TAACAGAG), but not pyrimidine, TATCCAT or TATCCAC boxes (Supplementary Table S3C and **Figure S2B**).

**Table 2:** Orthologs of rice and wheat α-amylase genes.

SF	Rice	Wheat			
		A	B	D	Unanchored
*amy1*	LOC_Os02g52700	6AL_amy1	6BL_amy2	6DL_amy1	
	LOC_Os02g52710	6AL_amy2	6BL_amy3	6DL_amy2	
	LOC_Os01g25510	6AL_amy3	6BL_amy4	6DL_amy3	
			6BL_amy5		
*amy2*	LOC_Os06g49970	7AL_amy1	7BL_amy1	7DL_amy1	Un_amy1
			7BL_amy2	7DL_amy2	Un_amy2
			7BL_amy3		Un_amy3
*amy3*	LOC_Os09g28400	5AL_amy1	5BL_amy1	5DL_amy1	
	LOC_Os09g28420				
	LOC_Os08g36900				
	LOC_Os08g36910				
*amy4*	LOC_Os04g33040	2AL_amy1	2BL_amy1	2DL_amy1	Un_amy4
	LOC_Os01g51754		3BL_amy1	3DL_amy1	

*Rice and wheat amy protein sequences were downloaded from ftp://ftp.ensemblgenomes.org/pub/plants/release-35/fasta/oryza_sativa/dna/ and ftp://ftp.ensemblgenomes.org/pub/plants/release-35/fasta/triticum_aestivum/dna/, respectively. They were aligned with barley amy protein sequences using the clustalW program (http://www.genome.jp/tools/clustalw/). Sequences in the same clads were regarded as the same subfamily (SF) members. A, B, and D: wheat A, B, and D genomes*.

### Wheat *amy* Gene Numbers and Conserved Motifs on Promoters

The number of *amy* genes in the wheat A, B, and D genomes was 6, 10, and 8, respectively. The other four *amy* genes are located in unsorted chromosomes (**Table 2**). The wheat B genome had the most *amy* genes with four, three, one and two members in subfamilies *amy1*, *amy2*, *amy3*, and *amy4*, respectively. The number of *amy1* and *amy2* genes in each wheat genome did not exceed those in the barley genome (**Table 2**). All of the wheat *amy2* genes had the same genomic arrangement as the barley *amy2* genes with three introns and four exons (Supplementary Table S2). Most of the wheat *amy1* genes had two introns and three exons except for 6BL4, Un1 and Un2, which had three or four introns. The protein sequence identities were high within the wheat amy1 or amy2, being >80% (Supplementary Tables S1C,D and **Figures S3**, **S4**). The sequence identities for promoters of *amy1* genes were 50–100%, but much lower for *amy2* gene promoters (Supplementary Tables S4D,E). The promoters of all wheat *amy1* genes contained GARE (TAACAAA), pyrimidine, TATCCAT or TATCCAC boxes (**Supplementary Figure S5**). They also had a cAMP-like motif (TGAGCTC) box as per the barley *amy1* gene promoters. Five of the wheat *amy2* gene promoters contained a GARE (TAACAGAG) box, six contained pyrimidine and TATCCAT boxes, and seven had O2S motifs. The O2S motifs in the wheat genomes contained four variable nucleotides between the conserved CTTC and TCATC (**Supplementary Figure S6**), while the O2S in the barley *amy2* promoters had two variable nucleotides (**Figure 3**).

### A Comparison of *amy* Gene Copy Numbers and Sequence Properties from Barley, Rice, and Wheat

Barley had the highest number of *amy1* genes (six), while wheat had four in the B genome and rice had three (**Table 2**). Both barley and wheat had the same number of *amy2* genes (B genome only), while rice had one. Rice contained the most *amy3* genes (four), while barley and wheat had one each. All the barley, wheat and rice genomes contained two *amy4* genes (**Table 2**). The intron numbers for *amy2* genes were the same for barley, wheat and rice, but differed for the *amy1* genes: barley had two, wheat had two or four, and rice had two or three (Supplementary Table S2). The intron numbers for *amy3* and *amy4* genes differed, ranging from two to nine (Supplementary Table S2). Barley *amy1* genes had high sequence identities with wheat *amy1* genes ranging from 83 to 97% (Supplementary Table S5). Barley *amy2* genes also had high sequence identities with wheat *amy2* genes (80–96%) (Supplementary Table S5). The barley and wheat *amy1* and *amy2* genes had similar promoter regions with sequence identities ranging from 50 to 76% (Supplementary Table S6). However, there was no similarity between barley and rice promoter sequences.

### Expanded *amy1* and *amy2* Genes Are Important for Barley Malting Qualities

Barley amy proteins are grouped into four subfamilies according to their sequence properties (Mascher et al., 2017). The biological functions for each subfamily are unclear. Genetic mapping using molecular marker technologies showed that the regions associated with the genetic markers amy1 and amy2 on chromosomes 6 and 7 are important for malting qualities including amy enzyme activities and malt extracts (Hayes et al., 1993; Han and Ullrich, 1994; Oziel et al., 1996; Marquez-Cedillo et al., 2000; Emebiri et al., 2004). Thus, we conclude that *amy1* and *amy2* are the major genes responsible for starch degradation during seed germination. Other indirect evidence includes the induced expression of *amy1* and *amy2* genes during seed germination by GA (Khursheed and Rogers, 1988; Karrer et al., 1991). The abundance of mRNA levels ranks amy32b (*amy2_3*) > amy6-4 (*amy1_1a* to *amy1_1e*, possibly sum) > amy46 (*amy1_2*) about 24 h after GA induction (Khursheed and Rogers, 1988; Karrer et al., 1991). Barley *amy1* have expanded members due to genome duplication, which may play a key role in barley becoming a malting commodity. Furthermore, many amy genomic and cDNA clones had been deposited on Genbank. Their relationships for some of the clones with amy1 and amy2 was shown by a phylogenetic tree at **Supplementary Figure S7**.

There was no direct evidence for the function of amy3 and amy4 proteins in barley. In wheat, amy3 was highly expressed in developing grains, which affected carbon partitioning and diacylglycerol accumulation (Whan et al., 2014), while the amy4 gene may be involved in starch degradation working in partnership with amy1 proteins (Mieog et al., 2017).

### Significance of Conserved Motifs on Promoters of *amy1* and *amy2* in the Induction of *amy* Gene Expression

Barley amy enzymes are synthesized in barley aleurone layers induced by gibberellin (GA) (Chrispeels and Varner, 1967; Jacobsen et al., 1970). Two groups of proteins (A and B) were detected after GA induction. Their responses to GA induction differed in a time and GA concentration dependent manner (Jacobsen and Higgins, 1982). The group A proteins expressed earlier and required a low GA concentration, while the group B proteins were not detectable till 8 h after GA addition and required a higher GA concentration. However, the synthesis of group B proteins accelerated once expressed, and one of group B isoforms was most abundant at 24 h (Jacobsen and Higgins, 1982). The group A and B proteins were likely to be the products of *amy1* and *amy2* genes, respectively, as shown by changes in mRNA levels in response to GA (Khursheed and Rogers, 1988). Conserved motifs on *amy1* and *amy2* promoters played key roles in the induction of gene expression. A comparison of *amy1* with *amy2* promoters showed substantial differences in nucleotide composition of the conserved motifs. The GA-responsive element is TAACAAA on all *amy1* promoters, but TAACAGAG on all *amy2* promoters for all three crops. There is a cAMY-like responsive element close to the pyrimidine box on barley and wheat *amy1* promoters, but not *amy2* promoters. The difference in the conserved motifs may play a key role in GA-induced gene expression.

Gene expression induced by GA may have the same mechanism for all *amy1* members, since they contain extract same number of motifs with same nucleotide sequences except *Hvamy1_2* gene on which the TATCCAC box was replaced by TATCCAT. In contrast, barley *amy2* members may be differentially regulated, particularly for the *Hvamy2_2* gene, since the conserved motifs differed substantially from *amy1*, *amy2_1*, and *amy2_3*. Furthermore, no similar motifs were found in the promoters of *amy3* and *amy4*.

### Genomic Properties of Wheat *amy* Genes Did Not Differ from Those of Barley *amy* Genes

Barley grains are often used for malting. Barley malts contain sufficient diastatic power (enzymatic hydrolytic activities) to completely convert starch to fermentable sugars. Extended numbers of *amy* genes and the presence of GA-regulatory motifs are important for barley to be used for malts (**Table 1** and **Figures 2**, **3**). However, wheat *amy* genes had similar genomic properties to barley *amy* genes with similar intron and exon structures (Supplementary Table S2). They also contained GA-regulatory motifs as in barley *amy* gene promoters (**Supplementary Figures S5**, **S6**). This could explain why wheat can also be used for malting (Fleming et al., 1960; Faltermaier et al., 2013). However, there are some problems with using wheat malts for brewing. Wheat grains lack husk, which is a problem for a brewing process called Lautering. Wheat also has higher protein (up to 20%). Wheat proteins promote foam formation, but also enhance haze issues (Faltermaier et al., 2015).

## Conclusion

Subfamilies *amy1* and *amy2* have similar genomic properties in wheat and barley—including the number of exon/intron structures, localized on chromosomes 6 and 7, with GA-responsive elements on promoters—but differ in rice. Interestingly, the barley genome contains at least three more *amy1* genes on chromosome 6H. Wheat should contain sufficient amy activity to completely degrade starch during malting. Other factors, such as high protein and the lack of husk, may limit the use of wheat for brewing.

## Author Contributions

All authors listed have made a substantial, direct and intellectual contribution to the work, and approved it for publication.

## Conflict of Interest Statement

The authors declare that the research was conducted in the absence of any commercial or financial relationships that could be construed as a potential conflict of interest.

## Footnotes

1 http://www.genome.jp/tools/clustalw/

2 http://www.ncbi.nlm.nih.gov/Structure/bwrpsb/bwrpsb.cgi

## Supplementary Material

FIGURE S1 Alignment of rice amy1 and amy2 proteins. Rice amy1 and amy2 proteins were aligned using ClustalW software (http://www.genome.jp/tools-bin/clustalw). The secondary structure features are shown on top of the alignments. There are three domains: domain A (black arrows and asterisks), domain B (green arrows) and domain C (red arrows). Domain A consists of a (α/β)_8_ barrel, while domain C has five β-sheets. The three catalytic amino acids Asp_203_, Glu_228_, and Asp_310_ are indicated by red asterisks.

FIGURE S2 Rice *amy1* and *amy2* promoter alignments. **(A)** ALL *amy1* contain a GA-responsive element (GARE) TAACAAA (red box), a pyrimidine box (CCTTTT) and a TATCCA(C/T) box (blue boxes). **(B)** Rice amy2 promoter (LOC_Os06g49970) within 500 bp from translation start codon (ATG) contained GA-responsive element only. No pyrimidine and TATCCA(C/T) were found.

FIGURE S3 Alignment of wheat amy1 proteins. Wheat amy1 proteins were aligned using ClustalW software (http://www.genome.jp/tools-bin/clustalw).

FIGURE S4 Alignment of wheat amy2 proteins. Wheat amy2 proteins were aligned using ClustalW software (http://www.genome.jp/tools-bin/clustalw).

FIGURE S5 Alignment of wheat amy1 promoter sequences. ALL wheat *amy1* contain a GA-responsive element (GARE) TAACAAA (red box), a pyrimidine box (CCTTTT), a TATCCA(C/T) box (blue boxes) and a cAMP-like motif (TGAGCTC) (green box).

FIGURE S6 Alignment of wheat *amy2* promoter sequences. The GA-responsive element (GARE) TAACAAA (red), pyrimidine box (CCTTTT), TATCCA(C/T) box (blue), and OS2 motifs (green) are marked.

FIGURE S7 A comparison of new and old amy nomenclatures. The phylogenetic tree was generated using the ClustalW program (http://www.genome.jp/tools-bin/clustalw). The nucleotide sequences were collected from GenBank with accession numbers M17125.1, M17126.1, M17127.1, and M17128.1 (Knox et al., 1987); X15226 and X15227 (Rahmatullah et al., 1989); J01236.1 (Rogers and Milliman, 1983); K02637 (Rogers, 1985); J04202 (Khursheed and Rogers, 1988), and X05166 (Whittier et al., 1987).
