# Supplementary figures and images for: Comparisons of Copy Number, Genomic Structure, and Conserved Motifs for α-Amylase Genes from Barley, Rice, and Wheat
Source: Front Plant Sci. 2017 Oct 5;8:1727. doi: 10.3389/fpls.2017.01727 (PMC5633601; doi:10.3389/fpls.2017.01727)

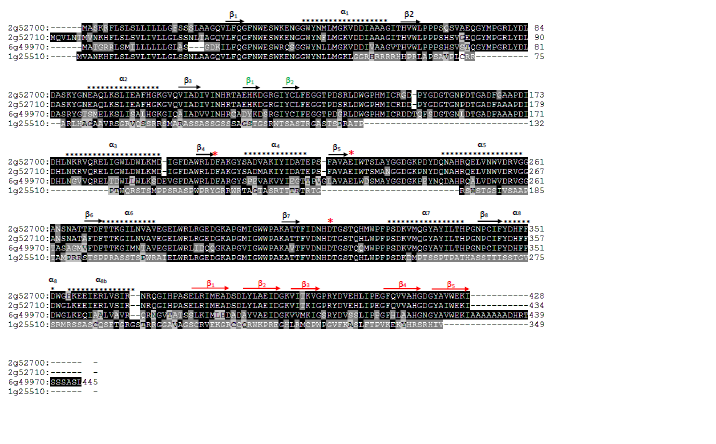

Supplement: FIGURE S1 — Alignment of rice amy1 and amy2 proteins. Rice amy1 and amy2 proteins were aligned using ClustalW software (http://www.genome.jp/tools-bin/clustalw). The secondary structure features are shown on top of the alignments. There are three domains: domain A (black arrows and asterisks), domain B (green arrows) and domain C (red arrows). Domain A consists of a (α/β)8 barrel, while domain C has five β-sheets. The three catalytic amino acids Asp203, Glu228, and Asp310 are indicated by red asterisks. [file Image_1.TIF]

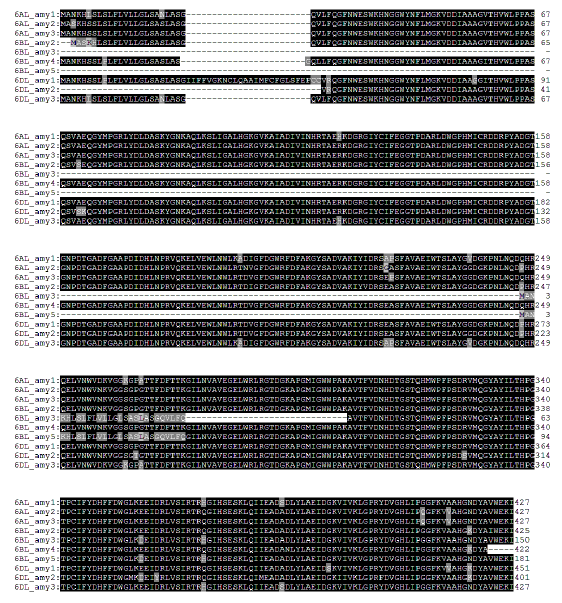

Supplement: FIGURE S3 — Alignment of wheat amy1 proteins. Wheat amy1 proteins were aligned using ClustalW software (http://www.genome.jp/tools-bin/clustalw). [file Image_3.TIF]

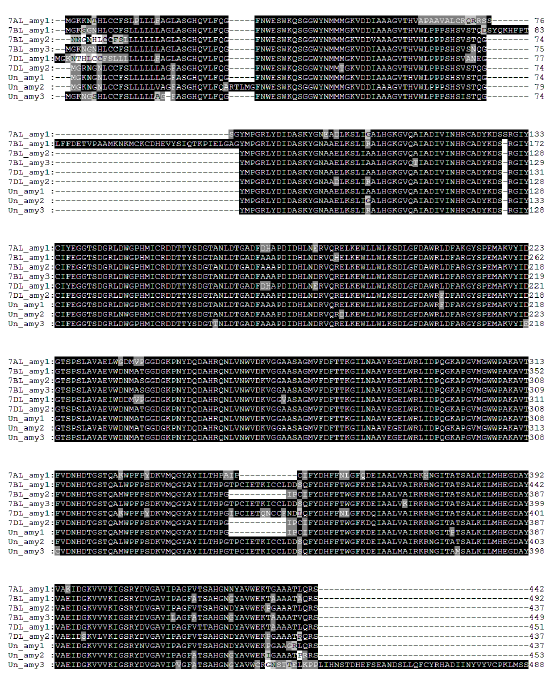

Supplement: FIGURE S4 — Alignment of wheat amy2 proteins. Wheat amy2 proteins were aligned using ClustalW software (http://www.genome.jp/tools-bin/clustalw). [file Image_4.TIF]

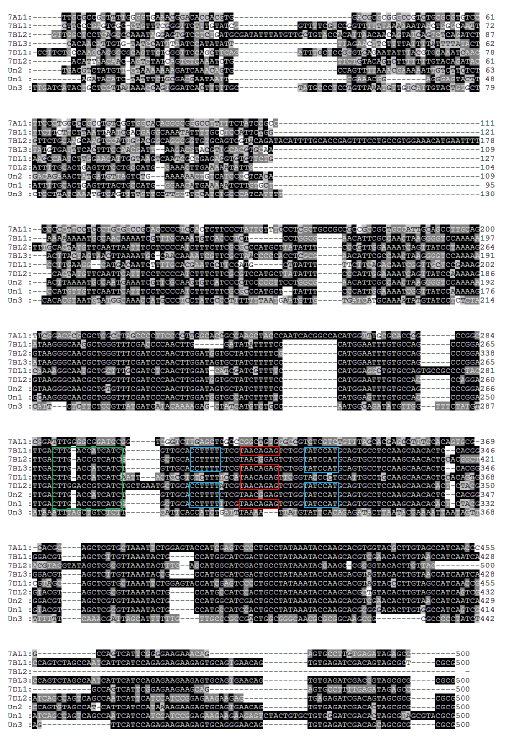

Supplement: FIGURE S6 — Alignment of wheat amy2 promoter sequences. The GA-responsive element (GARE) TAACAAA (red), pyrimidine box (CCTTTT), TATCCA(C/T) box (blue), and OS2 motifs (green) are marked. [file Image_6.TIF]
