# Supplementary material for: Comparisons of Copy Number, Genomic Structure, and Conserved Motifs for α-Amylase Genes from Barley, Rice, and Wheat
Source: Front Plant Sci. 2017 Oct 5;8:1727. doi: 10.3389/fpls.2017.01727 (PMC5633601; doi:10.3389/fpls.2017.01727)
Supplement: FIGURE S2 — Rice amy1 and amy2 promoter alignments. (A) ALL amy1 contain a GA-responsive element (GARE) TAACAAA (red box), a pyrimidine box (CCTTTT) and a TATCCA(C/T) box (blue boxes). (B) Rice amy2 promoter (LOC_Os06g49970) within 500 bp from translation start codon (ATG) contained GA-responsive element only. No pyrimidine and TATCCA(C/T) were found. [file Image_2.PDF]

A

```
2g52700:-------------TTCCTCCTTGCTGCACTCCAATGTAACGAATTAATCATATGTACTGTTTCTCTCTTTTAAAGAAATTCAGATTGGGA 77
2g52710:----------GTATGATTTTTTGCAAAAATTTTCTATAGG-AAAGTTACTTAAAAATTATATTAATCTATTTTTAAAATTTAAAATAGTT 79
1g25510:TTGTATTATCTTTACAGTGTTTTAAGCCTTTCAATATAAAAAAAAGGCCTTTCAACATATAGATAGACTTTTACAGACTTTGTCAAAATT 90

2g52700:AGCTTTCCCG--------CGTGACCGGTCTAAAAAAATATTTATTGATAAATGATGATAAAATATAATCGAGAACCAAACGCACCTGCAA159
2g52710:AATACTCAATTAATTATACGTTAATGGCTCAGCTCGTTTTGCGTACATTCTCAATCGATTCTTTTCCTCTGCTCTCAAATGCTCTG--TG167
1g25510:TGTAGTACTAAG---ATACGTCATATGCATCTAAGATCTCTTATAACATGGAGAGGGAGTAATTTACAAGG-------TCGCTCTT----166

2g52700:TGCGATCAGGTGTTTTAGCTTCAGCTCGCACAAGCACATGCAAGAAAGCCGGATTTCCTACTAACCGGCACCTTTTTGAGGGCTCCATCT249
2g52710:TGCGATCAGGTATTCATG-TTCAGCTCGCACAAGCACAAGCAAGACAGATGGAATTCCTACTGACCTGCGCCTTTTG-AGTGCTCCAACT255
1g25510:--CAGCAAAAAATTCATG-TTCAGCTCGCACAAGCACAAGCAAGAAAGATGGAATTCCTACTGACTTGTGCCTTTTG-AGTGCTCCATCT252

2g52700:CTCAAG--------GCCATTAAAGTGCCTATGGGCTCACCGGCCAATAACAAACT---GCTG-------TTATCCATCCAATCCAGTGTC321
2g52710:CTCAAAGTCTCAAGGCCATTAAATTGCCTATGGGCTCACCAGCCAATAACAAACTCCGGCTG-------TTATCCATCCAATCCAGTGTC338
1g25510:CTCAAG--------GCCATTAAATTGCCTATGGGCTCACCGGCCAATAACAAACTCCTACTGACCAGTGTTATCCATCCAATCCAGTGTC334

2g52700:CCAAAGCAACATTCAAGCTAGCCCAACAACCAGGCCTCCAAGAGTAGCAAGTTGAGCGTG---CAATCCCCGGCATT-CTCGACTATAAA407
2g52710:CCAAAGCAACATTCAAGC-------CCAGCCAGGCCTCCAAAAGTTGCAAGTTGAGCATGGCAAAATCCCCGGCAATTCTCGACTATAAA421
1g25510:CCAAAGCAACATTCAAGC-------CCAGCCAGGCCTCCAAAAGTTGCAAGTTGAGCATGGCAAAATCCCCGGCAATTCTCGACTATAAA417

2g52700:TACCCGACCAGAAACACACCCAG-CTTCATCACTCCATCTCGCCTTGTCTCATTCCAAGTATAGCTGCAGCCTGCACGTACTGTACTGAG496
2g52710:TACCTGACCAGACACACCCAGGAGCTTCATCAATCATCCATCTCCG----------AAGTGTGTCTGCAGCATGCAGGTGCTGAACACC-500
1g25510:TACCTCAGCAGACACACACCCAG-CTTCATCAATCCACCCACCTCCG---------AAGTGTATCTGCAGCATGCAGGTACCGATCGAAC497

2g52700:GACA500
2g52710:----   -
1g25510:ACA-500
```

B

ACGAATTAGTCCAAGAATATGGATGTGTTTTATTAATAGCCTACGTTTAATATTTATAATTAGTATCCAAACATTTAA
TGTGATAGGGAGTTAAAACTCAAGTTTTAGTCCCACCTAAACACGGTTCATCAGGATACAAGATATCAGCACAGTG
TTGATTGAGTAGGTTCATCAGGAAGCAGAGTAATAGCACGTAACAGAGTTGGGTATCCTGTATACAGCAGTTGTC
CCCAAGGAAGCAACTCTCCATCACATCACATCGCGGAATCTAAAAAAGAATTCATCAATCCTCCATTTTCTAAAAGA
AAAGGAAAGAATTCATCAATTCTGCCCTGCCGCAGCTGCGTCCTATAAATACCCTACACGCGTAGCCATCTTCTGCA
ATCATCAAAACACCTGTAATCATCTTCTGAATTCATCAATTCAGAGTTCAGAGCAGAGTGTCCCTGCTTCTGTGCAG
AGGAGAGAGATCGAGCTATAGAGCTAGCCTGGCTGCTGCC

Figure S2 Rice *amy1* and *amy2* promoter alignments

A: ALL *amy1* contain a GA-responsive element (GARE) TAACAAA (red box), a pyrimidine box (CCTTTT) and a TATCCA(C/T) box (blue boxes).

B: Rice amy2 promoter (LOC_Os06g49970) within 500 bp from translation start codon (ATG) contained GA-responsive element only. No pyrimidine and TATCCA(C/T) were found.
