# Supplementary material for: Comparisons of Copy Number, Genomic Structure, and Conserved Motifs for α-Amylase Genes from Barley, Rice, and Wheat
Source: Front Plant Sci. 2017 Oct 5;8:1727. doi: 10.3389/fpls.2017.01727 (PMC5633601; doi:10.3389/fpls.2017.01727)
Supplement: FIGURE S5 — Alignment of wheat amy1 promoter sequences. ALL wheat amy1 contain a GA-responsive element (GARE) TAACAAA (red box), a pyrimidine box (CCTTTT), a TATCCA(C/T) box (blue boxes) and a cAMP-like motif (TGAGCTC) (green box). [file Image_5.PDF]

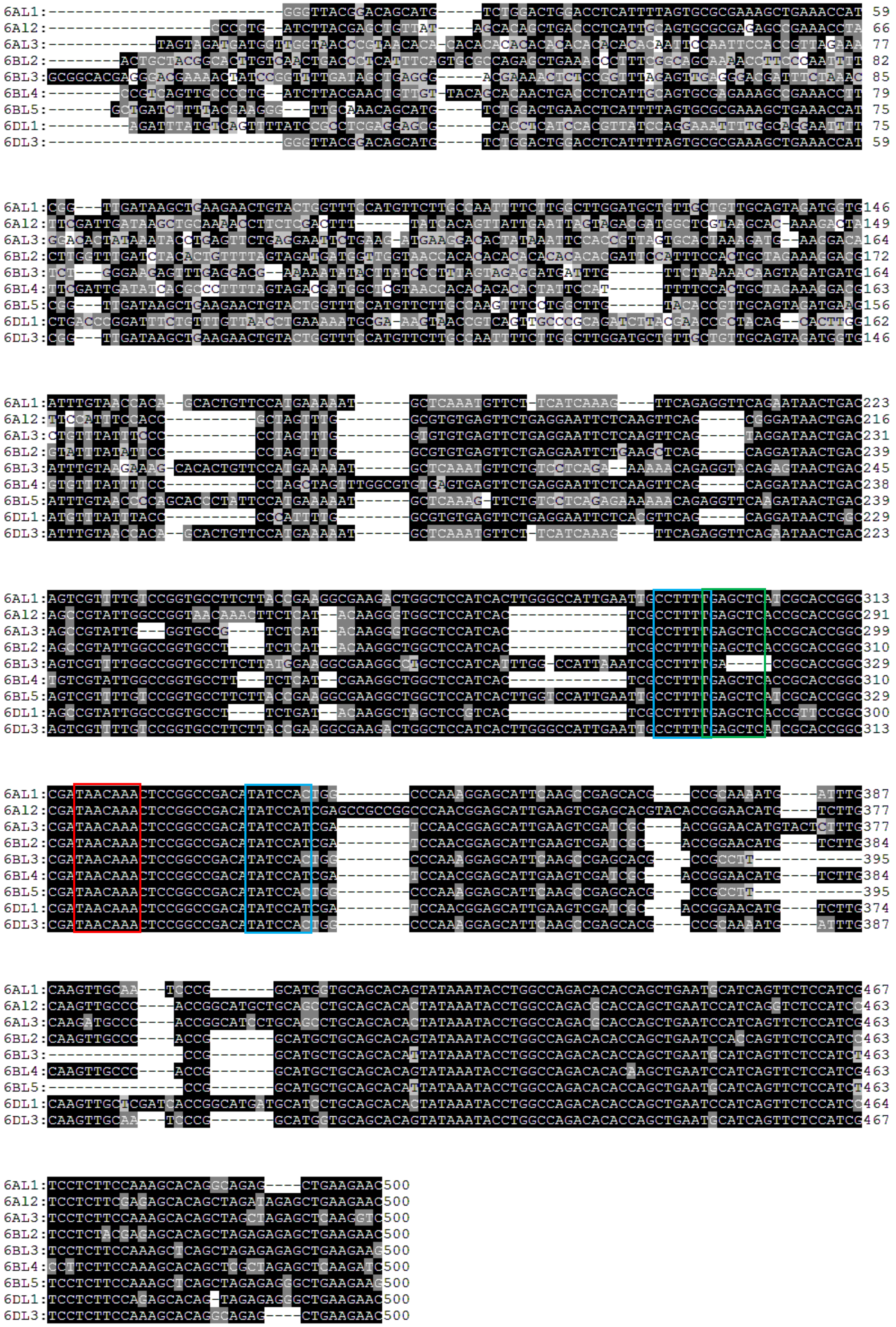
6AL1
6Al2
6AL3
6BL2
6BL3
6BL4
6BL5
6DL1
6DL3

Figure S5 Alignment of wheat amy1 promoter sequences

ALL wheat *amy1* contain a GA-responsive element (GARE) TAACAAA (red box), a pyrimidine box (CCTTTT), a TATCCA(C/T) box (blue boxes) and a cAMP-like motif (TGAGCTC) (green box).
