# Supplementary material for: Comparisons of Copy Number, Genomic Structure, and Conserved Motifs for α-Amylase Genes from Barley, Rice, and Wheat
Source: Front Plant Sci. 2017 Oct 5;8:1727. doi: 10.3389/fpls.2017.01727 (PMC5633601; doi:10.3389/fpls.2017.01727)
Supplement: FIGURE S7 — A comparison of new and old amy nomenclatures. The phylogenetic tree was generated using the ClustalW program (http://www.genome.jp/tools-bin/clustalw). The nucleotide sequences were collected from GenBank with accession numbers M17125.1, M17126.1, M17127.1, and M17128.1 (Knox et al., 1987); X15226 and X15227 (Rahmatullah et al., 1989); J01236.1 (Rogers and Milliman, 1983); K02637 (Rogers, 1985); J04202 (Khursheed and Rogers, 1988), and X05166 (Whittier et al., 1987). [file Presentation_1.PDF]

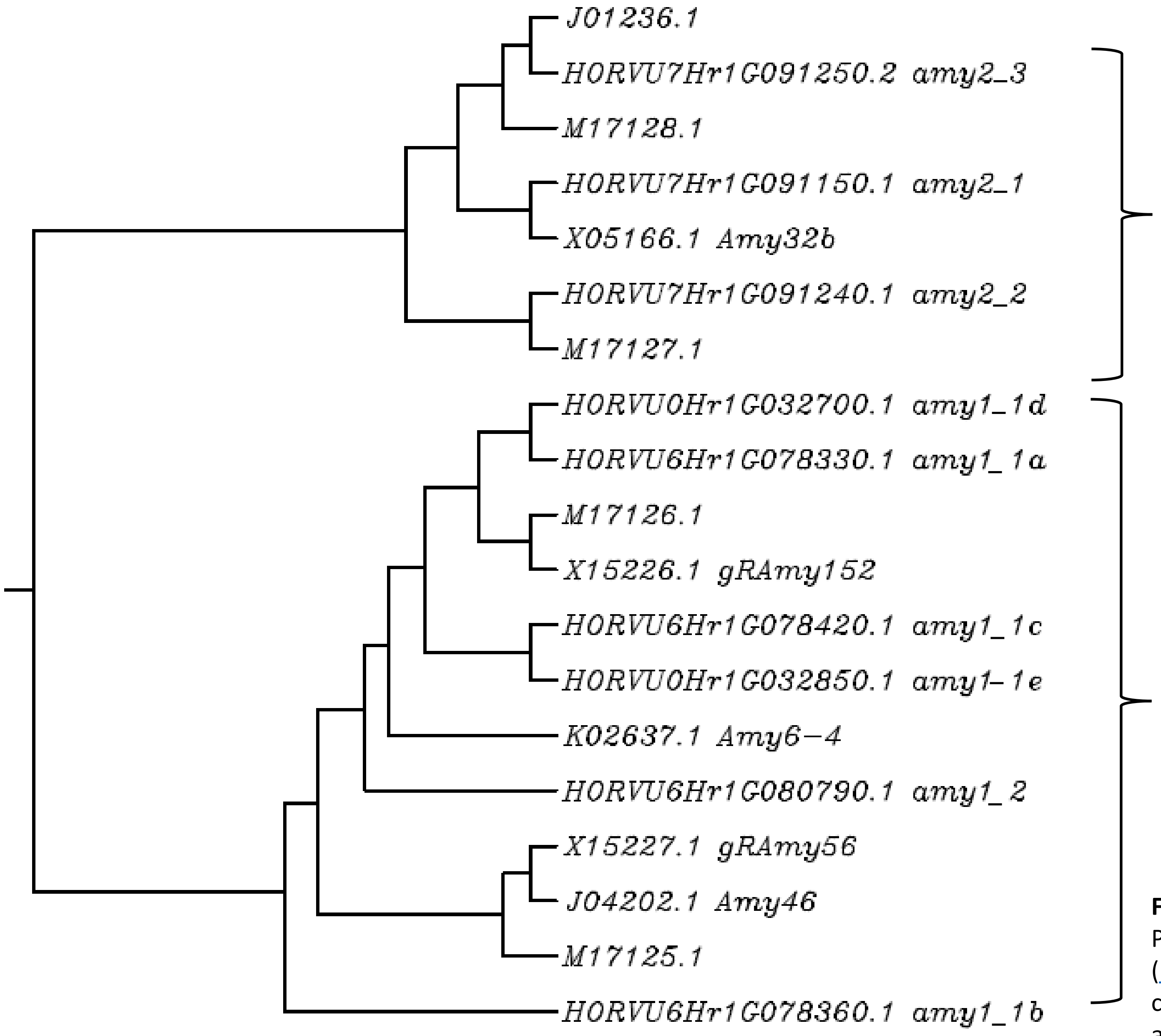

New nomenclatures
Amy Subfamily 2: amy2_1, amy2_2, amy2_3

Old nomenclatures
Type B, AMY1, α-amylase 2, Amy2, high pI α-amylase, Amy32b

New nomenclatures
Amy Subfamily 1: amy1_1a, amy1-1b, amy1_1c, amy1_1d, amy1_1e, amy1_2

Old nomenclatures
Type A, AMY2, α-amylase 1, Amy1, low pI α-amylase, Amy6-4, Amy46

Figure S7 A comparison of new and old amy nomenclatures
Phylogenetic tree was generated by ClustalW program (http://www.genome.jp/tools-bin/clustalw). The nucleotide sequences were collected from Genbank with accession numbers M17125.1, M17126.1, M17127.1 and M17128.1 (Knox et al., 1987); X15226 and X15227 (Rahmatullah et al., 1989); J01236.1 (Rogers and Milliman, 1983); K02637 (Rogers, 1985); J04202 (Khursheed and Rogers, 1988) and X05166 (Whittier et al., 1987).
